# Therapeutic Glypican‐3 CRISPR Genome‐Editing Using UltraLarge Porous Silica Nano‐Depot for the Treatment of Hepatocellular Carcinoma

**DOI:** 10.1002/smsc.202400447

**Published:** 2024-11-21

**Authors:** Sanghee Lee, Sian Lee, Hyojin Lee, Seongchan Kim, Dong‐Hyun Kim

**Affiliations:** ^1^ Department of Radiology Feinberg School of Medicine Northwestern University Chicago IL 60611 USA; ^2^ School of Biomedical Engineering Korea University Seoul 02841 Republic of Korea; ^3^ Biomaterial Research Center, Biomedical Research Institute Korea Institute of Science and Technology (KIST) Seoul 02792 Republic of Korea; ^4^ College of Pharmacy and Research Institute of Pharmaceutical Sciences Gyeongsang National University Jinju Gyeongsangnam‐do 52828 Republic of Korea; ^5^ Robert H. Lurie Comprehensive Cancer Center Northwestern University Chicago IL 60611 USA; ^6^ Department of Biomedical Engineering McCormick School of Engineering Northwestern University Evanston IL 60208 USA

**Keywords:** crispr‐cas9, gene therapy, glypican‐3, hepatocellular carcinoma, porous silica nanoparticles

## Abstract

Glypican‐3 (GPC3) is a key diagnostic marker and therapeutic target in hepatocellular carcinoma (HCC), interacting with Wnt and Hippo/YAP pathways related to cancer proliferation. Modulating GPC3 gene expression can induce liver cancer cell death by disrupting growth factor signaling, cell adhesion, and metabolic regulation. This study presents the development of a non‐viral ultralarge porous CRISPR‐Cas9 silica nano‐depot to perform targeted GPC3 genome editing for the treatment of HCC. The synthesized ultralarge porous silica nano‐depot (UPSND) encapsulates substantial CRISPR‐Cas9‐ribonucleoprotein complexes with a remarkable 98.3% loading efficiency. The UPSND‐mediated GPC3 CRISPR‐Cas9 therapy significantly suppresses cancer cell proliferation by modulating the Wnt and Hippo/YAP pathways. The efficiency of GPC3 gene deletion is observed to be 5.1‐fold higher than that of commercial lipid‐based GPC3 CRISPR‐Cas9 in both human and murine genes, with minimal off‐target effects. In vivo systemic administration of GPC3 Cas9‐RNP@UPSND resulted in preferential accumulation within hepatic tissues in orthotopic HCC mouse models, leading to complete tumor eradication and enhancing *T‐cell* tumor‐infiltration. Furthermore, the GPC3 CRISPR‐Cas9@UPSND treatment exhibits superior anti‐proliferative efficacy in tumor‐growth prevention compared to Codrituzumab, as evidenced by the analysis of Ki67 and GPC3 expression, along with serum GPC3 levels. These findings underscore the translational potential of the non‐viral UPSND nanoplatform‐based CRISPR GPC3 genome editing, offering a promising targeted therapeutic strategy for HCC treatment.

## Graphical abstract

**GA:** An effective therapeutic strategy of Glypican‐3 CRISPR gene editing is achieved by developing an ultralarge porous silica nano‐depot (UPSND) for hepatocellular carcinoma (HCC) therapy. The non‐viral UPSND effectively encapsulated Cas9‐ribonucleoproteins and precisely modulated gene expression, outperforming lipid‐based nanocarrier. Glypican‐3 deletion via UPSND leads to complete tumor eradication and enhanced T‐cell infiltration, showing strong potential as HCC therapy with translational prospects.

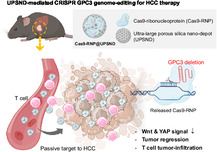

## 1 Introduction

Hepatocellular carcinoma (HCC) remains a significant challenge while being the fifth most common cancer worldwide.^[^ ^1^ ^]^ Surgical treatment and liver transplantation are currently the primary options, but they are only suitable for a limited number of patients diagnosed at an early stage with tumors smaller than 5 cm.^[^ ^2^ ^]^ The FDA‐approved chemotherapeutic agents for HCC, multitargeted tyrosine kinase inhibitors (e.g., sorafenib, lenvatinib, regorafenib, and cabozantinib), have limited effectiveness with adverse side effects, and the patients often develop resistance.^[^ ^3^ ^]^ Thus, developing new strategies for targeting or treating HCC liver cancers is more urgent than ever.

Glypican‐3 (GPC3), a heparan sulfate proteoglycan anchored to the cell membrane, has become a pivotal target for HCC therapy due to its overexpression in more than 70% of HCC tissues.^[^ ^4^ ^]^ GPC3 is highly expressed compared to alpha‐fetoprotein (AFP) and is not present in benign hepatic lesions or normal liver parenchyma, rendering it a more specific HCC biomarker. GPC3‐positive HCC is associated with poor prognosis and reduced survival rates.^[^ ^5^ ^]^ GPC3‐targeted imaging modalities, including positron emission tomography and magnetic resonance imaging (MRI) have demonstrated high specificity in the early diagnosis of HCC.^[^ ^4^, ^6^ ^]^ Additionally, GPC3 detection in the serum of many HCC patients further solidifies its value as a diagnostic marker.^[^ ^7^ ^]^ Beyond diagnosis, GPC3 is an attractive therapeutic target, with monoclonal antibodies such as Codrituzumab and chimeric antigen receptor‐T‐cell therapies.^[^ ^8^ ^]^ The five‐year survival rate for GPC3‐positive patients is significantly lower than for GPC3‐negative patients (54.5% vs. 87.7%, *p* = 0.031), underscoring the need for effective GPC3 inhibition strategies and further research on its role in HCC.[^5^, ^9^] Despite promising preclinical findings, clinical challenges such as incomplete GPC3 suppression, off‐target effects, and immune‐related adverse events complicate its therapeutic potential, necessitating further investigation.^[^ ^10^ ^]^

GPC3 alterations impact several signaling pathways within the tumor microenvironment (TME), regulating tumor cell proliferation and immune responses. Specifically, GPC3 activates the canonical Wnt pathway by directly interacting with the membrane receptor Frizzled through its unique chain, promoting HCC progression.^[^ ^6^ ^]^ Additionally, GPC3 modulates the Hippo signaling pathway, impacting cell–cell adhesion, organ size regulation, and tumorigenesis. GPC3 also interacts with the YAP pathway, promoting either oncogenic activity or tumor suppression, contingent on the specific cellular environment and context.^[^ ^11^ ^]^ Alterations in these pathways can impact immune cell migration, infiltration, and function, thereby modifying the immune response within the TME. Thus, reducing GPC3 function has been shown to enhance antitumor immune responses and affect tumor‐associated macrophage.^[^ ^12^ ^]^ However, current strategies using antibodies or RNA‐based knockouts may not fully eliminate GPC3 activity.

Recent advancements in CRISPR‐Cas9 gene‐editing technologies targeting EGFR, survivin, and Nrf2 genes offer promising potential for GPC3‐targeted HCC therapy.^[^ ^13^ ^]^ Ribonucleoproteins (RNP) complexes, composed of the Cas9 protein and a single guide RNA (sgRNA), provide significant advantages over mRNA‐based delivery. Pre‐assembled RNP bypasses the transcription and translation steps required for mRNA, enabling faster gene editing.^[^ ^14^ ^]^ RNP degrades quickly after use, reducing Cas9 activity duration and minimizing off‐target effects.^[^ ^15^ ^]^ This transient presence also lowers the risk of autoimmune responses and bioavailability issues compared to mRNA.^[^ ^16^ ^]^ Nevertheless, RNP delivery remains inefficient due to its large molecular size, underscoring the need for effective delivery systems. Lipid‐based carriers often struggle to encapsulate and protect large RNP effectively.^[^ ^17^ ^]^ In contrast, porous silica nanostructures offer several advantages, including biocompatibility, stability, uniform size, and sufficient internal space for encapsulating large complexes.^[^ ^18^ ^]^ Specifically, these porous structures have demonstrated the ability to encapsulate high molecular weight proteins such as β‐Gal, albumin, and RNase A.^[^ ^19^ ^]^ These capabilities, along with enhanced cellular uptake and targeted tissue delivery shown in previous studies, suggest they could maximize therapeutic efficacy over other non‐viral gene‐delivery carriers.^[^ ^20^ ^]^

**Figure 1:**
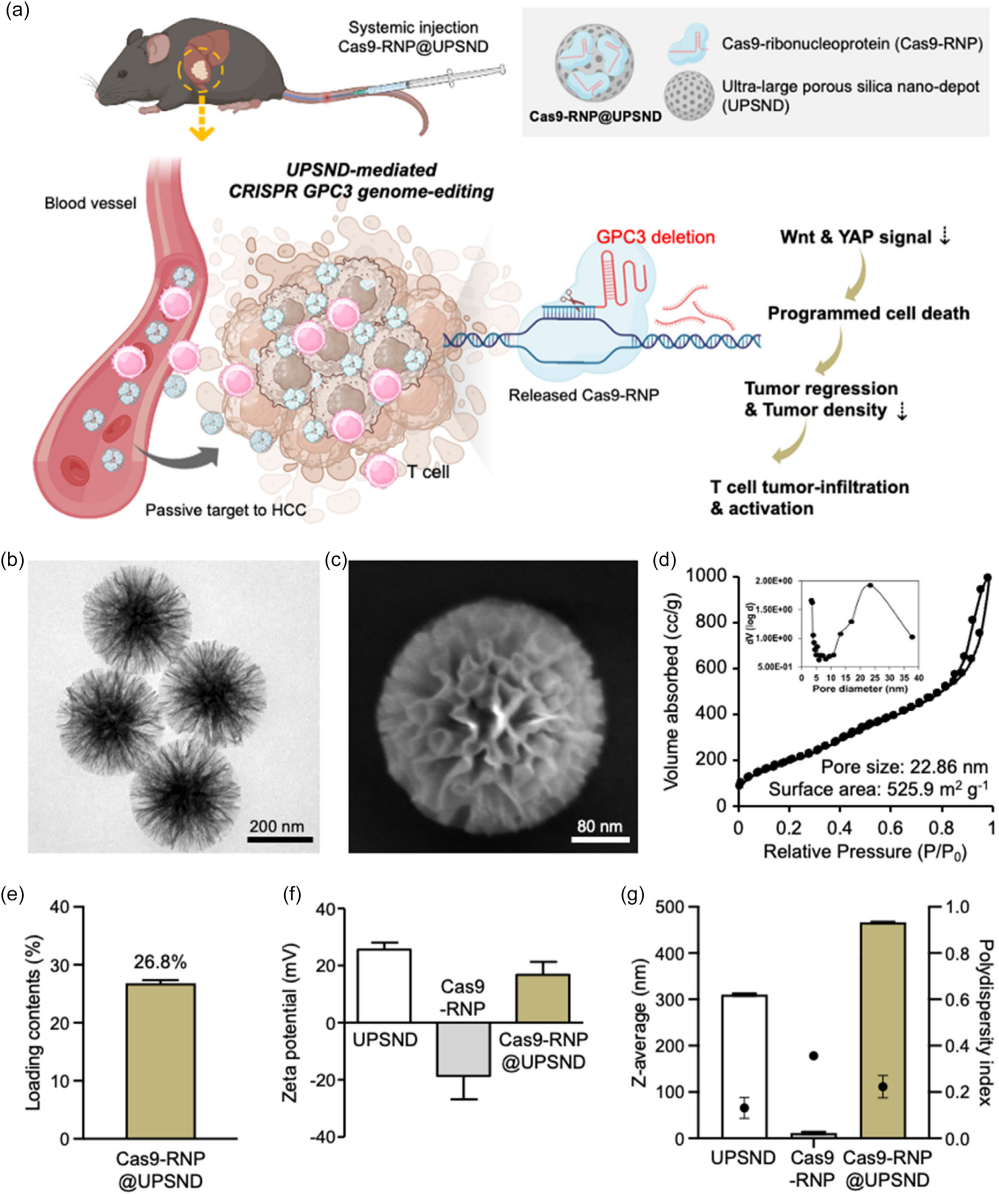
Preparation of UPSND and Cas9‐RNP complex. a) Schematic illustration of UPSND‐mediated Cas9‐RNP delivery to treat hepatocellular carcinoma (HCC). b) Representative transmission electron microscopy (TEM) and c) scanning electron microscopy (SEM) image of UPSND. Scale bars are 200 and 80 nm. d) Nitrogen absorption/desorption isotherms of UPSND for pore size and surface area. Inset: the Barrett–Joyner–Halenda pore size distribution curves. e) Loading contents of Cas9‐RNP into UPSND. f) Zeta‐potentials of UPSND, Cas9‐RNP, and UPSND‐loaded Cas9‐RNP (Cas9‐RNP@UPSND) (*n* = 3). g) Hydrodynamic size and polydispersity index measured using dynamic light scattering (DLS) analysis (*n* = 10). All data are presented as the mean ± s.d.

In the present study, we developed a non‐viral ultralarge porous CRISPR‐Cas9 silica nano‐depot to perform GPC3 genome editing for the treatment of HCC. The synthesized ultralarge porous silica nano‐depot (UPSND) showed enhanced Cas9‐RNP loading and delivery efficiency compared to the conventional vector, lipofectamine (Lipo). Our optimized Cas9‐RNP loaded UPSND (Cas9‐RNP@UPSND) demonstrated an enhanced anti‐cancer cell killing in various HCC cell lines. Then, we further investigated the effects of GPC3 deletion using Cas9‐RNP@UPSND on drug resistance and analyzed its impact on key regulatory molecules within the Wnt and Hippo/YAP pathways to understand the underlying mechanisms. Finally, the biological and therapeutic effectiveness of Cas9‐RNP@UPSND along with anti‐cancer T‐cell immunity were evaluated in orthotopic HCC mouse models (**Figure** **1**a). Our findings suggest that non‐viral UPSND‐mediated GPC3 CRISPR‐Cas9 knockout is a promising strategy for the treatment of HCC.

## 2 Results and Discussion

### 2.1 Characterization of UPSND and Cas9‐RNP@UPSND

UPSNDs offer an efficient platform for loading Cas9‐RNP due to their high surface area, large pore volume, and strong absorptive properties. The pore size of UPSND is critical for the efficient encapsulation and release of genetic materials, such as RNA, plasmid DNA, or Cas9‐RNP. For loading Cas9‐RNP, theoretical pore sizes should be over 10 nm to accommodate the large‐sized Cas9 protein (≈10 × 10 × 5 nm) and the sgRNA complex (≈100 nucleotides).^[^ ^21^ ^]^ Moreover, optimally sized pores can protect Cas9‐RNPs from enzymatic degradation in physiological environments, ensuring their stability and efficacy during the in vivo delivery process.^[20a]^ Transmission electron microscopy (TEM) and scanning electron microscopy (SEM) images showed the morphology of the 300 nm UPSND (Figure 1b,c and Figure S1a,b, Supporting Information). The nitrogen sorption analysis showed that the prepared UPSND had an average pore size of 22.86 nm and a surface area of 525.9 m^2^ g^−1^. (Figure 1d). According to our experiments, the porosities are particularly suitable for efficient loading, release, and protection of the Cas9‐RNP. The Cas9‐RNP loaded into the UPSND (Cas9‐RNP@UPSND) was created by mixing Cas9‐RNP and UPSNDs in phosphate‐buffered saline (PBS, pH 7.4) at room temperature (RT) for 30 min. The loading contents of Cas9‐RNP within the UPSND, quantified via a bicinchoninic acid (BCA) assay, were determined to be 26.8% (w/w) (Figure 1e), representing the highest reported content among those of previously reported porous nanoparticle‐based RNP delivery systems to date (e.g., zeolitic nanoparticles, porous silica nanoparticles, hydrogel).^[^ ^22^ ^]^ After loading the Cas9‐RNP into the UPSND, we observed changes in the zeta potentials and hydrodynamic size. The positively charged UPSND, with a zeta potential of 24.2 ± 0.7 mV, showed a decrease due to electrostatic interactions with the negatively charged Cas9‐RNP (−18.6 ± 8.3 mV). This indicates that 16.8 ± 4.5 mV of Cas9‐RNP were complexed with the UPSNDs (Figure 1f). In addition, the hydrodynamic size of the Cas9‐RNP@UPSND increased to ≈466.5 ± 1.2 nm, compared to the original UPSND (310.3 ± 2.5 nm) and the Cas9‐RNP (11.6 ± 2.4 nm) (Figure 1g and Figure S1c, Supporting Information). These results suggest that the Cas9‐RNP were effectively encapsulated within the pore of UPSND while maintaining their overall surface characteristics and size. To evaluate the loading efficiency (LE) of the Cas9‐RNP, we compared UPSND to Lipo, a widely used lipid‐based transfection reagent. Under optimal conditions, UPSND achieved an LE of 98.3% for sgRNA and 98.3% for Cas9, while Lipo exhibited an LE of 47.8% for sgRNA and 51.9% for Cas9 (Figure S2, Supporting Information). These results demonstrate that UPSND can efficiently encapsulate Cas9‐RNP due to its ample internal space and strong electrostatic interactions. This suggests a potential for enhanced delivery efficiency into cells or target organs and the eventual release of the cargo. Moreover, proper interspace can influence the release of the Cas9‐RNP and its stability. The behavior of the encapsulated Cas9‐RNP was further evaluated by examining the release profiles from UPSND and Lipo. After 16 h, ≈69.5% of sgRNA remained encapsulated within UPSND, compared to only 23.4% retention in Lipo. Similarly, 98.5% of Cas9 was initially loaded into UPSND, with 61.9% remaining after 16 h, compared to 18.2% release from Lipo (Figure S2, Supporting Information). The demonstrated high Cas9‐RNP loading capacity and sustained release, facilitated by the optimized internal porosity and electrostatic properties of UPSND, underscore its superior potential as an advanced delivery system for achieving precise and effective CRISPR‐Cas9 gene editing.

### 2.2 Targeted GPC3 Genome‐Editing in Human HCC Cells

**Figure 2:**
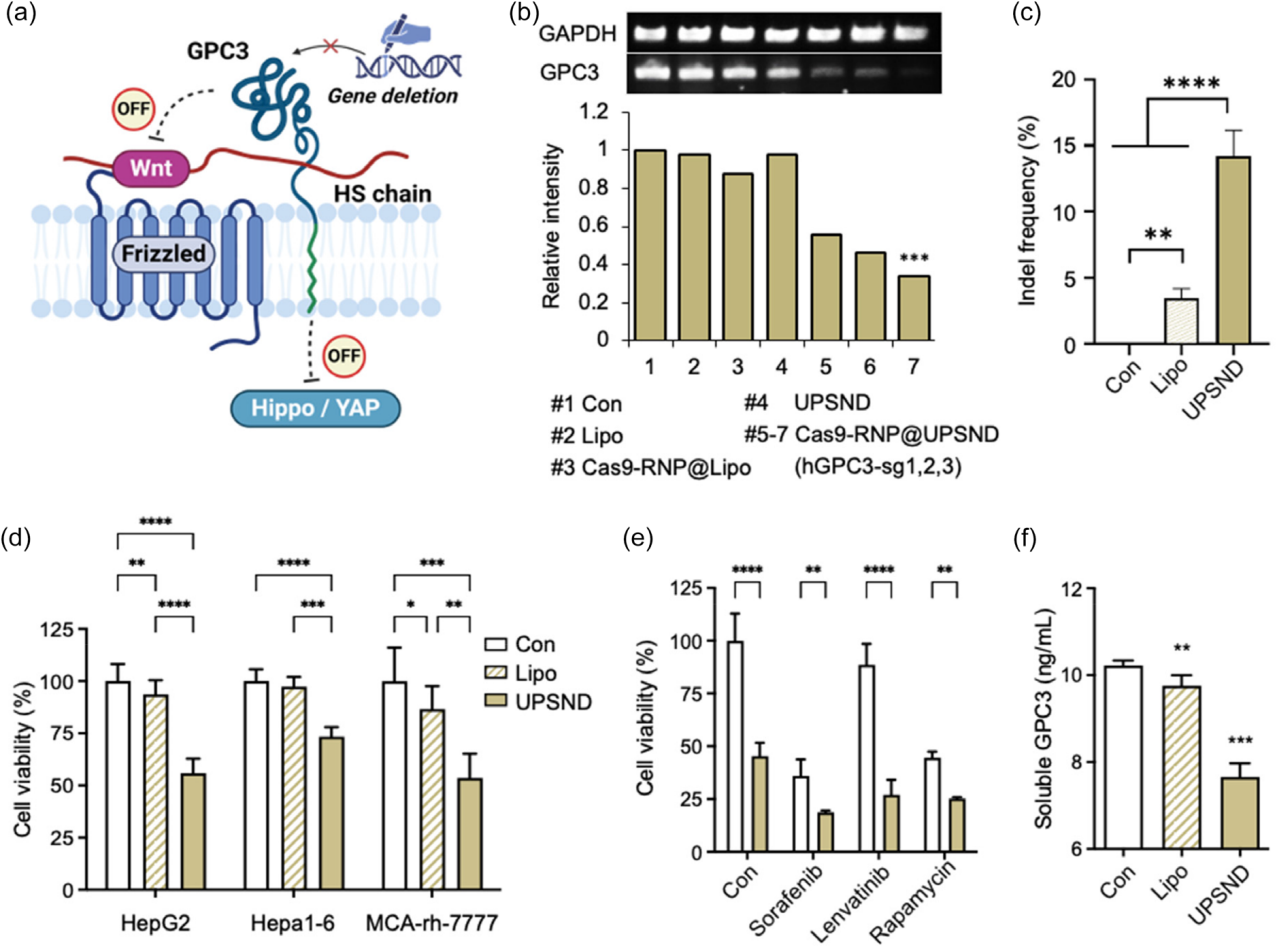
Genome editing efficacy in human HCC cells. a) Schematic illustration of GPC3 deletion and the subsequent biological mechanisms involving the Wnt and Hippo/YAP signaling pathways. b) Gene‐editing efficacy as determined by RT‐PCR. Relative expression levels were quantified from the band intensities of the control group using image J software (*n* = 3). c) Analysis of the gene deletion frequency of GPC3 in HepG2 cells by next‐generation sequencing (NGS) (*n* = 3). d) Multicellular assessment of GPC3 gene editing using hGPC3 sgRNA sequences (*n* = 8). e) Evaluation of tyrosine kinase inhibitor drug efficacy post‐GPC3 gene editing in HepG2 cells (*n* = 3). f) Released soluble GPC3 levels in cell supernatant (*n* = 6). All data are presented as the mean ± s.d. P‐values: ns, not significant, *p < 0.0332, **p < 0.0021, ***p < 0.0002, and ****p < 0.0001.

GPC3 is a crucial regulator of several cell signaling pathways, including Wnt and Hippo/YAP, and plays significant roles in cell growth and proliferation. Cleavage of GPC3 decreases Wnt and YAP activities, potentially leading to cell death (**Figure** **2**a). However, the importance of GPC3 cleavage in tumor development remains unclear.

To identify potential sgRNA sequences for cleaving the GPC3 gene in human HCC, an in‐silico analysis was conducted to evaluate predicted gene‐editing efficiency and off‐target effects. From the initial pool, three sgRNA candidates (hGPC3‐sg1, sg2, and sg3) were selected based on their optimal targeting efficiency and minimal off‐target potential (Figure S3a, Supporting Information). The final candidates are highlighted, demonstrating the criteria used for selection. Among these, hGPC3‐sg3 exhibited the highest gene‐knockout efficiency, reducing mRNA levels by up to 67.2%, compared to hGPC3‐sg1 (45.5%) and hGPC3‐sg2 (54.1%) as determined by reverse transcription‐polymerase chain reaction (RT‐PCR) (Figure 2b).

For subsequent experiments, hGPC3‐sg3 was utilized to edit the GPC3 gene in human cells to assess its impact on inhibiting liver cancer cell growth. We tested cell viability at different UPSND‐to‐Cas9‐RNP ratios and found that GPC3 gene editing significantly inhibited HCC cell growth, with the optimal ratio being 1:1, resulting in 55% cell viability (Figure S3b,c,d, Supporting Information). Following two days after Cas9‐RNP@UPSND treatment, we extracted genomic DNA from the cell populations and performed targeted deep sequencing using Illumina MiniSeq (Table S1, S2, Supporting Information). The results revealed a mutation rate of 14.8%, which is five times higher than the mutation rates obtained with Cas9‐RNP@Lipo (Figure 2c). The functionality of this optimized ratio was confirmed across multiple HCC cell lines, with the most efficient editing observed in human HepG2 cells (Figure 2d). Additionally, the hGPC3‐sg3 sequence not only targeted human GPC3 but also showed activity in mouse Hepa1‐6 and rat MCA‐rh‐7777 cell lines. Furthermore, cell viability with UPSND‐mediated delivery was ≈55.9% higher than with Lipo‐mediated GPC3 gene editing (93.7%). The impact of different material concentrations (ranging from 0 to 640 μg mL^−1^) on cell biocompatibility was also examined, revealing that over 90% of cells remained viable across all concentrations of UPSND, whereas cells treated with Lipo maintained viability at less than 25% (Figure S4, Supporting Information). These results indicate the biocompatibility of UPSND itself and highlight the effective cell death induced solely by UPSND‐mediated Cas9‐RNP delivery.

GPC3‐mediated pathways contribute to drug resistance by changing the TME and cellular signaling. GPC3 expression influences the anticancer efficacy of tyrosine kinase inhibitors (TKIs) in HCC by impacting Wnt/β‐catenin and Hippo pathways.^[^ ^23^ ^]^ Although TKIs do not directly target GPC3 genes, their expression can modify the TME and cellular signaling, thereby affecting drug sensitivity. It was assumed that reducing GPC3 expression could restore the sensitivity of resistant HCC cells to TKIs. The results indicate that GPC3 knockout enhances TKI effectiveness. GPC3 gene‐edited cells showed a 27.0% reduction in viability after Lenvatinib treatment, compared to an 88.6% reduction in the control group, which displayed significant resistance at the same concentration (Figure 2e). Similarly, Sorafenib and Rapamycin treatments significantly improved therapeutic outcomes in GPC3 gene‐edited cells. These findings suggest that combining GPC3‐targeting strategies with TKIs could enhance therapeutic outcomes by addressing multiple pathways involved in tumor survival and therapeutic resistance.

Additionally, membrane‐bound GPC3, anchored to the cell membrane, is cleaved by specific enzymes to produce its soluble form.[^12^, ^24^] As membrane‐bound GPC3 acts as a precursor for soluble GPC3, editing the membrane‐bound form can affect the production of soluble GPC3. UPSND‐mediated GPC3 gene editing reduced the levels of soluble GPC3, implying a potential impact on the role of GPC3 in HCC growth and associated signaling pathways (Figure 2f). Since soluble GPC3 is a well‐known serological marker essential for the early detection of HCC,^[^ ^25^ ^]^ cleaving GPC3 could be a novel therapeutic approach for treating HCC.

### 2.3 Modulation of Wnt and Hippo/YAP Signaling Pathways

**Figure 3:**
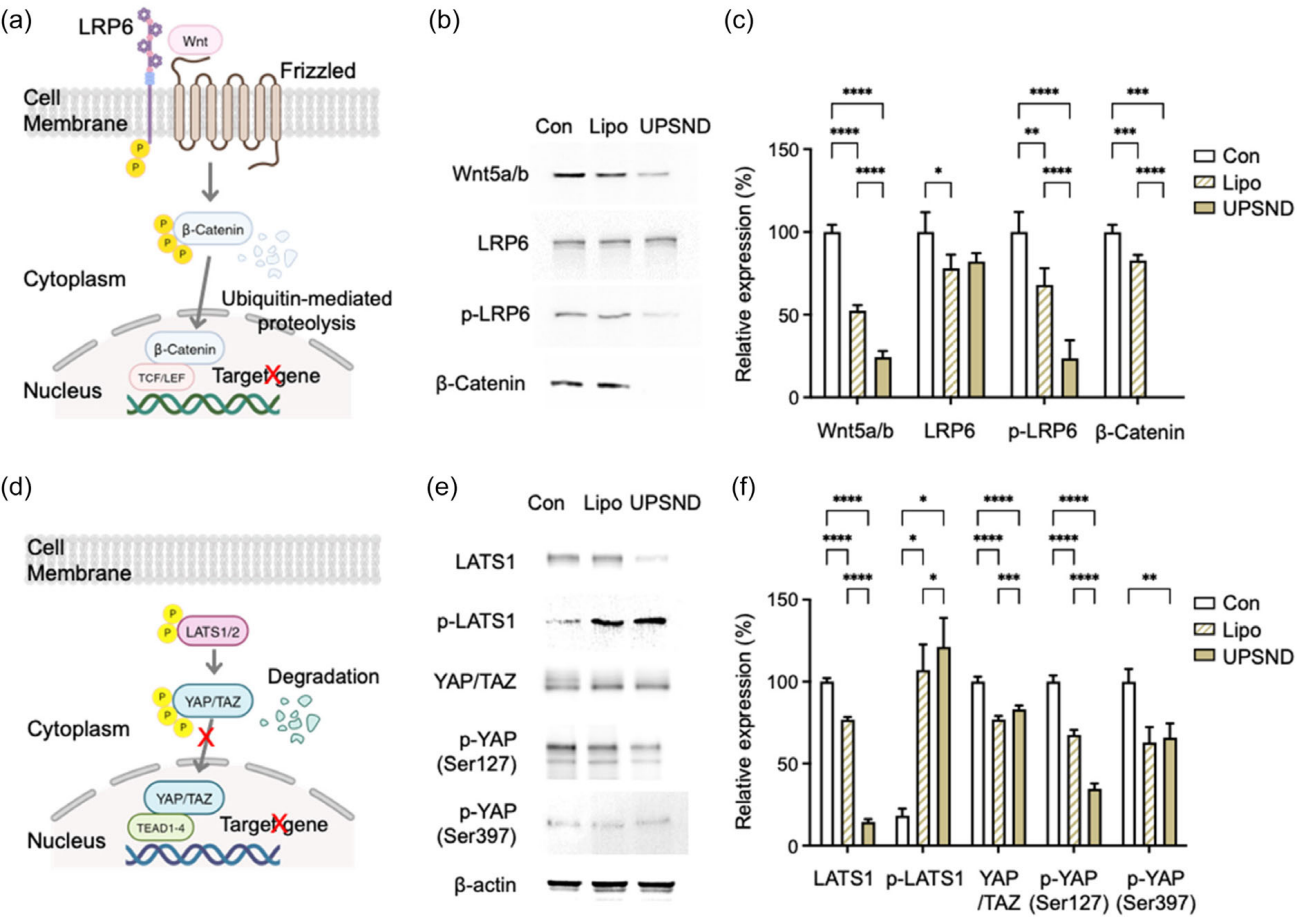
Modulation of Wnt and Hippo/YAP signaling pathways resulting from GPC3 gene editing in human HCC cells. a–c) Wnt signaling pathway induced by UPSND‐mediated GPC3 gene editing. Immunoblots were performed for key molecules, including Wnt5a b^−1^, LRP6, phosphorylated (p)‐LRP6, and β‐Catenin. d–f) Hippo/YAP signaling changes induced by UPSND‐mediated GPC3 gene editing. Immunoblots were performed for key molecules, including p‐LATS1, YAP/TAZ, p‐YAP (Serine 127), and p‐YAP (Serine 397). Relative expression levels were quantified from the band intensities normalized to ß‐actin using Fiji software (*n* = 3). All data are presented as the mean ± s.d. P‐values: ns, not significant, *p < 0.0332, **p < 0.0021, ***p < 0.0002, and ****p < 0.0001.

To analyze the effects of GPC3 gene editing at the molecular level, the Wnt and Hippo/YAP signaling pathways were examined. In the group where the GPC3 gene was edited using Cas9‐RNP@UPSND, we observed a significant 0.2‐fold decrease in the levels of Wnt5a b^−1^ proteins, which are important components of the non‐canonical Wnt signaling pathways. This suggests that disrupting this pathway could have broader effects beyond canonical Wnt signaling. Additionally, the band corresponding to β‐catenin, a key downstream effector of the canonical Wnt pathway, disappeared after GPC3 gene editing. The expression and phosphorylation of low‐density lipoprotein receptor 6 (LRP6), an important Wnt co‐receptor, were also significantly affected, indicating disruption of the Wnt signaling pathway (**Figure** **3**a–c).

The Hippo/YAP pathway was also examined, which is closely linked to the Wnt signaling pathway.^[^ ^26^ ^]^ GPC3 gene editing via Cas9‐RNP@UPSND resulted in a 0.1‐fold reduction in the expression of large tumor suppressor (LATS) and a 6.6‐fold increase in the phosphorylation of LATS (p‐LATS), indicating enhanced Hippo pathway activity. This activation was further supported by a significant reduction in YAP/TAZ expression (0.8‐fold), as well as decreased levels of phosphorylated YAP (p‐YAP) at Ser127 (0.3‐fold) and Ser397 (0.7‐fold). Typically, unphosphorylated YAP translocates from the cytoplasm to the nucleus to promote gene transcription associated with tumor cell proliferation and survival. However, GPC3 gene editing may prevent this translocation, leading to the retention and degradation of YAP in the cytoplasm (Figure 3d–f). In summary, UPSND‐mediated Cas9‐RNP targeting of the GPC3 gene effectively disrupts both the Wnt and Hippo/YAP pathways. This dual impact has the potential to inhibit tumor growth and proliferation, offering a promising therapeutic gene therapy strategy for HCC.

### 2.4 Liver Tumor‐Specific Distribution of UPSND

**Figure 4:**
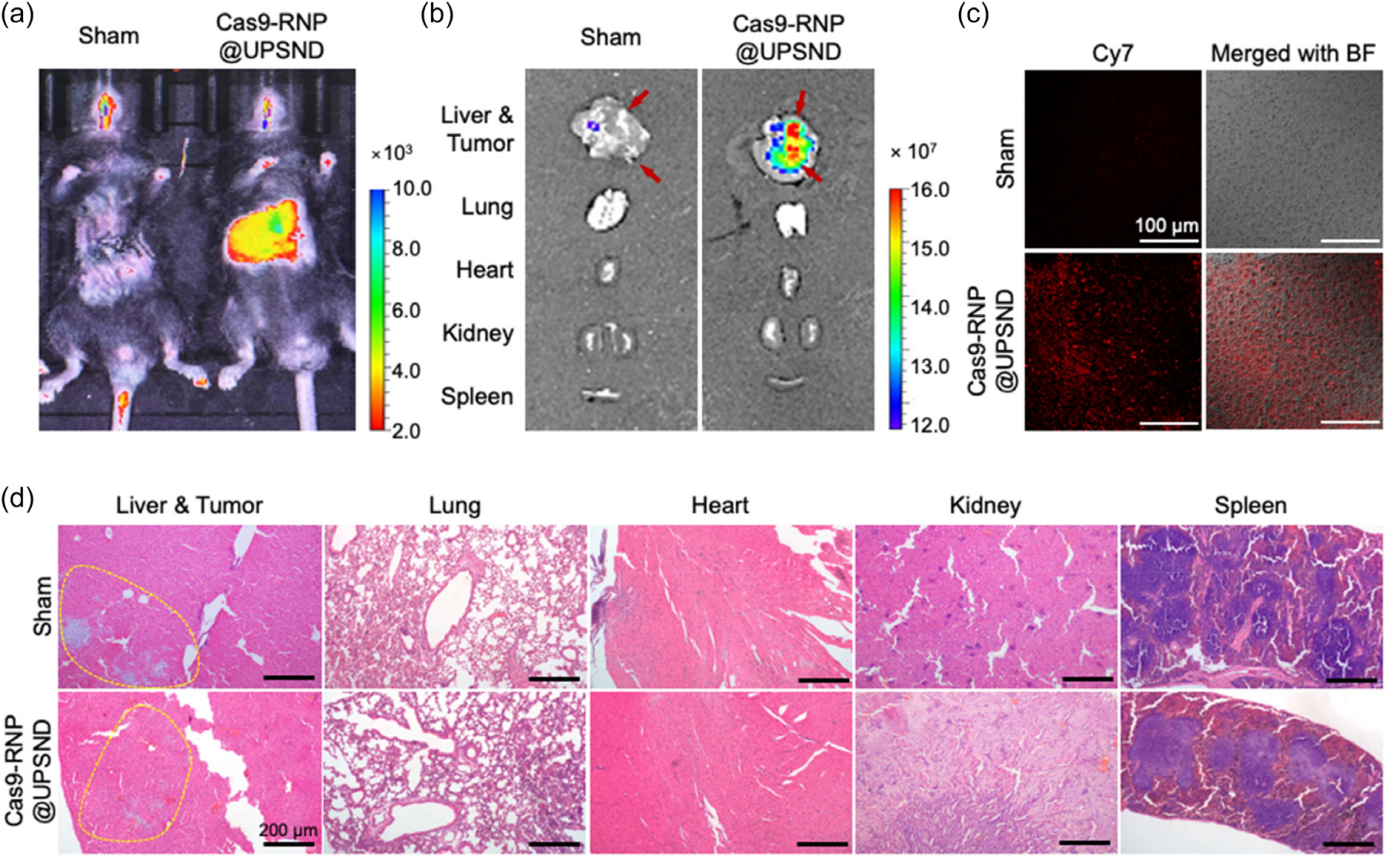
In vivo biodistribution in Hepa1‐6 orthotopic murine HCC models at 24 h post‐administration. Images of a) whole‐body and b) excised tissue after intravenous injection of Cas9‐RNP@UPSND. The fluorescence signals of the UPSND were strongly observed in the liver treated with Cas9‐RNP@UPSND (*n* = 3). c) The fluorescence images show a notable intensity of UPSND (Cy7, red) in sectioned liver tissue treated with Cas9‐RNP@UPSND. The fluorescence images were merged with bright field (BF) images. d) Hematoxylin and eosin (H&E) staining images of tumors and major tissues after treating Cas9‐RNP@UPSND. Intensive apoptotic cells are marked with yellow dashed lines.

Given the effectiveness of gene disruption and combinatorial kinase inhibitor mediated by Cas9‐RNP@UPSND in multiple liver cancer cell lines, we further expanded the application of Cas9‐RNP@UPSND delivery in a mouse tumor model. An orthotopic mouse HCC model was prepared by injecting Hepa1‐6 cell suspensions into the left lateral lobe of the liver, with tumor establishment monitored using T_2_‐weighted MRI (Figure S5a, Supporting Information). To monitor the distribution of Cas9‐RNP@UPSND after systemic injection, UPSND was labeled with Cy7 to minimize autofluorescence interference. Fluorescence was monitored by whole‐body imaging after intravenous injection of Cas9‐RNP@UPSND containing sgRNAs targeting GPC3, and major organs were subsequently excised from the mice. We found that the fluorescence signal of the Cas9‐RNP@UPSND was strongly observed in liver tumors, with negligibly detectable signals in other organs (**Figure** **4**a,b). The Cas9‐RNP@UPSND delivery took advantage of passive targeting with the enhanced penetration and retention effect, surface charge characteristics, and active cellular uptake mechanisms.^[^ ^27^ ^]^ According to previous studies, relatively larger nanoparticles (>200 nm) tend to accumulate more rapidly in the liver and spleen,^[^ ^28^ ^]^ and positively charged silica nanostructures exhibit an increased liver accumulation compared to unmodified nanostructures.^[^ ^29^ ^]^ Our results also demonstrate that Cas9‐RNP@UPSND primarily accumulates in the liver and HCC tumor sites, while it may also distribute slightly in other organs. Additionally, analysis of sectioned tumor tissues confirmed substantial tumor accumulation of Cas9‐RNP@UPSND, with a pronounced fluorescent signal localized in the cytoplasm (Figure 4c). Histological examination of major organs revealed no noticeable systemic toxicity (Figure 4d). Moreover, we developed UPSND‐doped with tantalum (Ta) to enable real‐time computed tomography (CT) imaging (Figure S5b, Supporting Information). Phantom studies showed that Ta‐doped UPSND provided an enhanced contrast in CT scans, indicating effective visualization. This capability supports the use of Ta‐doped UPSND in theragnostic applications, allowing for precise monitoring of gene delivery and therapeutic interventions. Taken together, these results indicate that UPSND enables the safe delivery of Cas9‐RNP and promotes tumor‐specific gene modification while minimizing any potential side effects in normal tissues.

### 2.5 Efficacy of GPC3 Genome‐Editing in Orthotopic HCC Mouse Models

**Figure 5:**
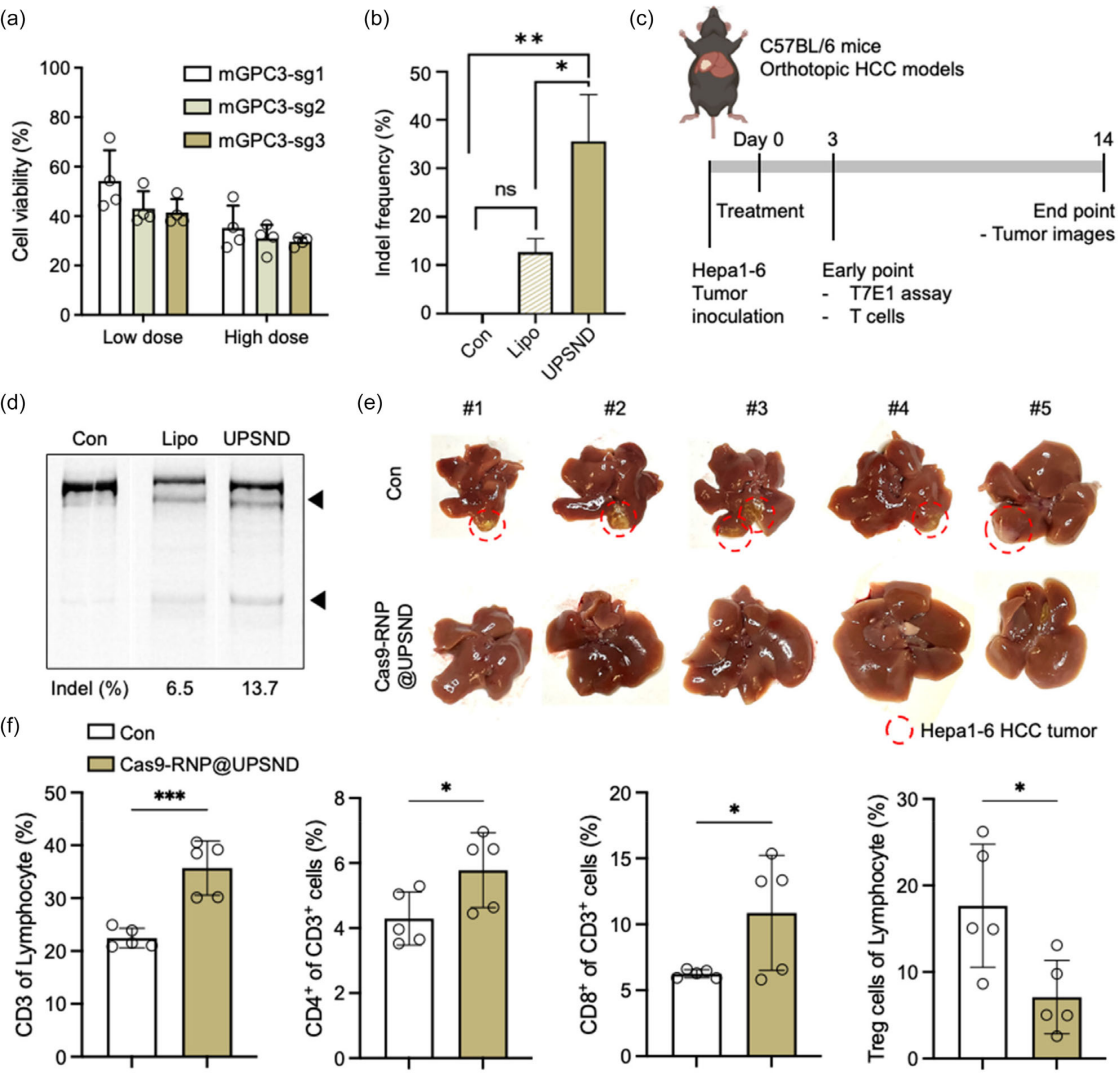
In vivo GPC3 genome editing in Hepa1‐6 orthotopic murine HCC models. a) Cell viability following UPSND‐mediated mGPC3 gene editing at varying sgRNA concentrations with a constant UPSND‐to‐sgRNA ratio (*n* = 4). b) Analysis of the gene deletion frequency of GPC3‐targeted Cas9‐RNP in Hepa1‐6 cells by NGS (*n* = 3). c) Experimental schedule. d) T7E1 indel analysis of GPC3 gene editing in Hepa1‐6 HCC tumors, with tumors harvested on day 3 post‐gene editing. e) Optical images of mouse liver tissues and Hepa1‐6 HCC tumors, with tumors indicated by red circles. f) T‐cell analysis following UPSND‐mediated GPC3 gene editing (*n* = 5). T lymphocytes (CD45^+^CD3^+^) are presented as the relative percentage of lymphocytes (CD45^+^). The helper *T* cells (Th, CD45^+^CD3^+^CD4^+^), cytotoxic *T* cells (Tc, CD45^+^CD3^+^CD8^+^), and regulatory *T* cells (Treg, CD45^+^CD3^+^CD4^+^CD25^+^Foxp3^+^) cells are presented with the relative percentage of T lymphocytes (CD45^+^CD3^+^). All data are presented as the mean ± s.d. P‐values: ns, not significant, *p < 0.0332, **p < 0.0021, ***p < 0.0002, and ****p < 0.0001.

To evaluate the tumor regression effects of UPSND‐mediated GPC3 gene editing, sgRNA sequences targeting mouse GPC3 were designed. Following a similar in silico analysis as for human sgRNA, we identified three potential sgRNA sequences for mouse GPC3 in murine HCC (mGPC3‐sg1, sg2, and sg3), predicting editing efficiency and minimized off‐target effects (Figure S6a, Supporting Information). Among these candidates, mGPC3‐sg3 demonstrated the most significant reduction in cell viability, with Hepa1‐6 cells showing 29.6% viability compared to mGPC3‐sg1 (35.2%) and mGPC3‐sg2 (31.0%) (**Figure** **5**a). Targeted deep sequencing analysis for bulk cell populations revealed a mutation rate of ≈ 35.6% for GPC3, which is three times higher than the mutation rates obtained with Cas9‐RNP@Lipo (Figure 5b and Table S1, S2, Supporting Information).

Tumor regression effects were explored using orthotopic Hepa1‐6 murine HCC models. Once tumors reached ≈20 mm^3^, Cas9‐RNP@UPSND was administered systemically every three days for a total of three doses (Figure 5c). GPC3 gene‐editing efficiency was confirmed by T7E1 assay on day 3 (Figure 5d and Figure S6b, Supporting Information). After 14 days, tumors were completely regressed in the UPSND‐mediated GPC3 gene‐editing group, whereas orthotopic tumors persisted in all control group mice (Figure 5e).

Previous studies have demonstrated that GPC3 blockade with monoclonal antibodies can disrupt tumor‐mediated immunosuppression, enhancing T‐cell activation and tumor infiltration by producing effector molecules like IFN‐γ and granzyme B.^[^ ^30^ ^]^ Based on this, we investigated T‐cell activation using tumor‐derived immune cells on day 14 (Figure 5f). In the GPC3 gene‐editing group, the expression of T‐cell receptors on lymphocytes increased 1.6‐fold, indicating a reduction in immune‐suppressive signals. GPC3 knockout likely facilitated T‐cell infiltration by removing barriers that typically prevent *T* cells from entering HCC tumors, resulting in enhanced activation of both helper *T* cells (Th, CD3^+^CD4^+^, 1.3‐fold) and cytotoxic *T* cells (Tc, CD3^+^CD8^+^, 1.7‐fold). The absence of GPC3, often implicated in immune evasion, boosted T‐cell receptor signaling and co‐stimulatory pathways, thereby promoting a more robust anti‐tumor response. Additionally, GPC3 loss also contributed to a 0.4‐fold reduction in regulatory T (Treg) cells, which otherwise inhibits effector T‐cell function within the TME. These findings suggest that Cas9‐RNP@UPSND‐mediated GPC3 knockout could enhance T‐cell‐mediated tumor eradication by promoting greater T‐cell infiltration and activation, thus improving antitumor immunity.

### 2.6 Prophylactic Potential of GPC3 Genome‐Editing in HepG2‐Bearing HCC Models

**Figure 6:**
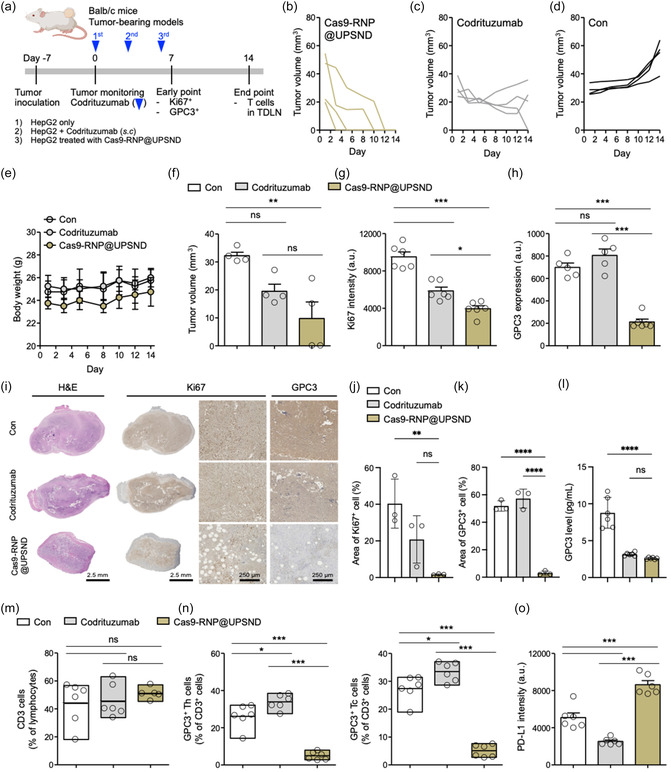
Prophylactic GPC3 gene editing in HepG2‐bearing HCC models. a) Experimental schedule showing specific time points. b–d) Individual tumor volume graphs for 14 days post‐GPC3 gene editing. e) Changes in body weight over 14 days. f) Average tumor volume on day 7. g) Ki67‐ and h) GPC3‐stained tumor cells were analyzed on day 7 using flow cytometry. i) Representative immunohistochemistry analysis on day 14. Tumor tissues were stained with H&E, Ki67, and GPC3. Scale bars are 2.5 mm and 250 μm, respectively. j,k) Quantification of positively stained tumor cells using Fiji software (*n* = 3). l) Serum GPC3 levels on day 14 (*n* = 5). Analysis of tumor‐draining lymph node immune cells on day 14 (*n* = 6). m) T lymphocytes (CD45^+^CD3^+^) are presented as the relative percentage of lymphocytes (CD45^+^). n) Th and Tc cells double positive for GPC3 are presented as the relative percentage of T lymphocytes (CD45^+^CD3^+^). o) PD‐L1 expression in tumor cells (CD45^−^PD‐L1^+^) was analyzed by fluorescent intensity. All data are presented as the mean ± s.d. P‐values: ns, not significant, *p < 0.0332, **p < 0.0021, ***p < 0.0002, and ****p < 0.0001.

Gene therapies are being researched for both the treatment of existing cancers and the prevention of cancer, particularly in individuals with a high genetic predisposition, as part of precision medicine approaches. In HCC, mutations in genes such as TP53 and PTEN are known to increase the risk of cancer development.^[^ ^31^ ^]^ Theoretically, correcting these mutations early with CRISPR‐Cas9 gene‐editing tools could potentially help prevent HCC. To explore this possibility, we conducted preventive gene editing in animal models by targeting and eliminating GPC3 expression in hepatocytes to prevent tumor formation.^[^ ^32^ ^]^ In HepG2‐bearing HCC models, we evaluated the in vivo gene‐editing efficiency of hGPC3 and compared the therapeutic effects of UPSND‐mediated GPC3 gene editing with those of anti‐GPC3 blockade (Codrituzumab), which has been tested in clinical trials. The study included three groups: 1) HepG2 cells injected into the flank without further treatment, 2) HepG2 cells injected followed by subcutaneous Codrituzumab, and 3) HepG2 cells treated with Cas9‐RNP@UPSND injected into the flank (**Figure** **6**a). HepG2 cells treated with Cas9‐RNP@UPSND showed reduced tumor establishment, with tumors disappearing in 2 out of 4 mice within 1 week and in the remaining mice within 2 weeks (Figure 6b). In contrast, Codrituzumab initially inhibited tumor growth but tumors generally maintained their volume, with only one tumor disappearing (Figure 6c). The tumors of the control group continued to grow (Figure 6d). Notably, UPSND‐mediated GPC3 gene editing did not significantly affect body weight, indicating minimal off‐target effects and no major disruption to metabolic or physiological functions (Figure 6e). On day 7, the average tumor volume in the GPC3‐edited group was 30.3% of that in the control group (Figure 6f).

Expression of Ki67, a marker of cell proliferation, was reduced by 0.4‐fold in the GPC3 gene‐editing group and by 0.3‐fold in the GPC3 blockade group (Figure 6g). This suggests that both strategies effectively disrupted cell proliferation and cell cycle progression. GPC3 expression was significantly reduced in tumor cells in the GPC3 gene‐editing group compared to the other groups, even though tumor proliferation was reduced in both groups (Figure 6h). Immunobiological analysis supported these results, showing lower densities of tumor cells and reduced Ki67 and GPC3 expression in the GPC3‐edited tumors (Figure 6i). Ki67‐positive tumor cells were only 1.7% in the GPC3‐edited group, compared to 20.8% in the Codrituzumab group and 40.3% in the control group (Figure 6j). Similarly, GPC3‐positive tumor cells were only 3.0% in the GPC3‐edited group, compared to 57.3% in the Codrituzumab group and 51.9% in the control group (Figure 6k). On the contrary, GPC3 blockade reduced serum‐soluble GPC3 levels by 0.3‐fold (Figure 6l), consistent with the in vitro result (Figure 2f). These results suggest that UPSND‐mediated GPC3 gene editing is more effective in preventing tumor formation than Codrituzumab. This approach significantly reduces tumor proliferation and effectively decreases GPC3 expression within tumors. Our findings highlight the advantages of targeted gene editing over traditional antibody‐based therapies and demonstrate its potential for improving cancer treatment outcomes. This provides a strong foundation for further development and clinical application of this platform in cancer therapy.

Finally, we investigated T‐cell responses from tumor‐draining lymph nodes to gain deeper insight into immune cell activation and proliferation following GPC3 gene editing. Although xenograft models have limitations in capturing immune responses, we assessed T‐cell activation in response to specific antigens. The overall proportion of *T* cells, including activated Th and Tc cells, remained similar across the groups (Figure 6m). To identify GPC3 antigen‐specific *T* cells, we labeled them with the GPC3 protein and analyzed the co‐stimulation of GPC3 with its specific *T* cells. As a result, the proportion of GPC3‐positive Th and Tc cells was significantly lower in the GPC3 gene‐editing group, indicating successful deactivation of GPC3 (Figure 6n). Specifically, GPC3‐positive Th cells comprised 5.0% of *T* cells, compared to 33.9% in the Codrituzumab group and 26.2% in the control group. Similarly, GPC3‐positive Tc cells made up 5.1% of *T* cells, compared to 33.5% in the Codrituzumab group and 27.4% in the control group. Additionally, GPC3 appears to be involved in the tumor's immune evasion strategies, as the downregulation of GPC3 in cancer cells might affect PD‐L1 expression. Our findings showed that GPC3 gene editing in HCC led to a 1.7‐fold upregulation of PD‐L1, likely as a compensatory mechanism to maintain immune evasion (Figure 6o). Since GPC3 is involved in immune regulation, its loss might trigger the upregulation of other immune checkpoints, allowing the tumor to shield itself from immune activation.^[^ ^33^ ^]^ However, the interaction between GPC3 and PD‐L1 expression may be influenced by various factors and could vary depending on the specific tumor context, necessitating further experimental validation.

## 3 Conclusion

Our study demonstrates the robust therapeutic potential of UPSND‐based GPC3 CRISPR‐mediated gene editing in HCC. This work emphasizes a dual strategy: refining genetic modulation platforms for GPC3 and developing advanced nanocarriers for gene delivery. Unlike conventional RNA‐based approaches that merely reduce GPC3 protein levels, our CRISPR‐mediated approach targets the full excision of the GPC3 gene, paving the way for a more durable and effective therapeutic intervention against HCC. RNA‐based methods, such as microRNA, siRNA, and long non‐coding RNAs, have shown utility in reducing GPC3 expression and inhibiting HCC progression.^[^ ^34^ ^]^ However, these methods are hampered by persistent residual gene expression and the necessity for continuous administration of prodrugs due to their transient inhibitory effects. The UPSND‐based platform not only surpasses the limitations of existing nanocarriers, such as lipid‐based nanoparticles, polymeric nanoparticles, exosomes, and gold nanoparticles, but also provides significant advantages in genetic material stability and bioavailability. Each of these alternative carriers encounters substantial challenges, including rapid clearance by the immune system, manufacturing complexities, and issues related to cytotoxicity, biocompatibility, and gene‐delivery efficacy.^[^ ^35^ ^]^ In contrast, our UPSND‐based system overcomes these barriers with an added ability to degrade into non‐toxic byproducts, addressing long‐term safety concerns.^[^ ^36^ ^]^ Furthermore, the UPSND surface can be functionalized with contrast agents like tantalum, enabling real‐time CT image‐guided precision medicine.^[^ ^37^ ^]^

Our experimental results reveal that UPSND‐mediated CRISPR‐Cas9 delivery leads to a remarkable increase in gene deletion probabilities in both human HepG2 cells (5.1‐fold) and murine Hepa1‐6 cells (3.2‐fold) compared to Lipo‐based methods. This elevated efficacy was corroborated through comprehensive in vitro assays, including RT‐PCR, deep sequencing, cell viability tests, soluble GPC3 quantification, and analysis of downstream regulatory biomolecules. Additionally, we validated the in vivo GPC3 CRISPR gene‐editing capabilities with UPSND in two distinct animal models: an orthotopic mouse model and a mouse model harboring human HCC cells. UPSND‐loaded with mouse sgRNA, upon systemic administration, specifically accumulated in the liver, leading to complete tumor elimination and enhancing immune cell infiltration into the TME. UPSND‐loaded with human sgRNA not only demonstrated effective in vivo gene editing in human HCC but also, when compared to monoclonal antibody drugs targeting GPC3, revealed that complete gene excision offers superior tumor suppression compared to mere physical blockade. Overall, this study establishes UPSND nanocarriers as a robust platform for CRISPR‐mediated gene editing, presenting a promising strategy for the development of more effective and durable genomic therapies for HCC.

## 4 Experimental Section

4.1

4.1.1

##### Materials

All reagents used in the experiments were purchased from commercial sources without further purification. Cetyl trimethyl ammonium bromide (CTAB) was purchased from Acros Organics (MA, USA). 3‐aminopropyltriethoxysilane (APTES), toluene, 1‐butanol, tetraethyl orthosilicate (TEOS) were purchased from Sigma‐Aldrich (MO, USA). Dulbecco's modified Eagle's medium (DMEM), alpha minimum essential medium (αMEM), fetal bovine serum (FBS), penicillin‐streptomycin, Lipofectamine 2000 transfection reagent, and radioimmunoprecipitation (RIPA) buffer were purchased from Thermo Fisher Scientific (Gibco, Thermofisher, MA, USA). CCK‐8 assay kit was obtained from Dojindo (MD, USA). Ultrapure water was obtained from a Milli‐Q system. TRIzol regent for total RNA isolation and Superscript II reverse transcriptase was purchased from Life Technologies (CA, USA). Target sgRNAs (hGPC3‐sg1, hGPC3‐sg2, hGPC3‐sg3, mGPC3‐sg1, mGPC3‐sg2, and mGPC3‐sg3) were designed by online tools (http://crispr.mit.edu/ and http://chopchop.cbu.uib.no/). Primers and Cas9 protein were purchased from Bioneer (Korea). sgRNAs were purchased from Integrated DNA Technologies Inc. (Korea). Codrituzumab (HY‐P99013) was purchased from MedChemExpress (NJ, USA).

##### Preparation and Characterization of UPSND

1.2 g of urea and 1.89 g of CTAB were dissolved in 60 mL of deionized water under stirring at 70 °C for 1 h. Subsequently, toluene:1‐butanol:TEOS (20:1:1 v v^−1^) were quickly added to the reaction mixture and then further stirred at 75 °C for 12 h to promote the formation of the UPSND. After aging for 12 h at RT, products were isolated by centrifugation, followed by washing with deionized water and ethanol to remove any residual reactants. For surface functionalization with a positive charge, the dried UPSNDs were suspended in a solution of APTES and subjected to reflux at 120 °C overnight. The pore size, surface area, and pore volume of the synthesized UPSNDs were determined via nitrogen adsorption/desorption isotherms using a NOVA surface area analyzer (Nova 2200e, Quantachrome Instruments). Prior to measurement, the samples were degassed at 573 K for 12 h to remove any adsorbed impurities. The morphology of the UPSNDs was examined using TEM (Tecnai) and SEM (FE‐SEM, Inspect F50). The zeta potential of the particles was measured using a Zetasizer NS90 (Malvern Panalytical Ltd, Malvern).

##### Characterization of Cas9‐RNP@UPSND

For loading Cas9‐RNP to the UPSND, Cas9 protein and sgRNA were mixed for 5 min at RT to form the Cas9‐RNP, and the complexes were mixed with UPSND‐dissolved PBS for 1 h at RT. To confirm the loading of Cas9‐RNP onto the UPSND, the changes of zeta potential and hydrodynamic size were measured by Zetasizer Nano ZS90 (Malvern, UK). To monitor the release of sgRNA and Cas9 protein, the Cas9‐RNP were incubated in PBS for 4 or 16 h, and the supernatant was collected by centrifuge. The released sgRNA was detected by UV‐vis NanoDrop (Thermofisher, MA, USA), and the released Cas9 protein was measured by BCA assay (Thermofisher, MA, USA). LC and LE were calculated by the following formula: LC = (weight of the Cas9 protein or sgRNA in the UPSND)/(total weight of the Cas9‐RNP@UPSND) ×100 and LE =(weight of the Cas9 protein or sgRNA in the UPSND)/(feeding weight of the Cas9‐RNP@UPSND) ×100.

##### Cell Culture

HepG2 (human hepatocellular carcinoma, HB‐8065), Hepa‐1c1c7 (mouse hepatoma, CRL‐2026), and Hepa1‐6 (mouse hepatoma, CRL‐1830) were purchased from American Type Culture Collection (ATCC, VA, USA). Cells were cultured as monolayers in DMEM high (HepG2 and Hepa1‐6) or αMEM (Hepa‐1c1c7) supplemented with 10% heat inactivated FBS and 1% penicillin/streptomycin. Cells were cultured at 37 °C with 100% humidity and 5% CO_2_ and sub‐cultured in new media every 1–2 days.

##### Cytotoxicity

Cells were seeded in 96‐well (1 × 10^4^ cells per well) or 24‐well (5 × 10^4^ cells per well) culture plates. After 24 h, the cells were treated UPSND, Cas9‐RNP@UPSND, or Cas9‐RNP@Lipo for 6 h and incubated with fresh growth medium for 48 h according to the indicated conditions. The CCK‐8 assay was performed according to the manufacturer's instructions, and the absorbance was detected at 450 nm by spectrophotometer (Synergy HT, BioTek, VT, USA). For the confirmation of TKI resistance, cells were incubated with Cas9‐RNP@UPSND for 24 h, after that, treated Sorafenib (5 μM), Lenvatinib (5 μM), or Rapamycin (5 nM) for 4 h. After 24 h, cell viability was observed using CCK assay.

##### Western Blot

HepG2 cells (1 × 10^6^ cells per well) were seeded in 6‐well culture plates. After 24 h, the cells underwent GPC3 gene editing. The edited cells were digested using RIPA buffer, and obtained cell lysate using a centrifuge at 12 000 × g for 15 min at 4 °C. The protein dissolved supernatant was quantified using BCA assay according to the manufacturer's instructions. Protein 20 μg was separated by electrophoresis in Tris‐glycine UPSNDS buffer and was transferred to polyvinylidene difluoride membrane in Tris‐glycine native buffer. The membranes were blocked with EveryBlot blocking buffer (#12 010 020, Bio‐rad, CA, USA) to inhibit nonspecific binding and analyzed using the indicated primary and secondary antibodies. Blots were reacted with enhanced chemiluminescence buffer and detected using Azure300 (Azure biosystems, CA, USA).

##### Targeted Deep Sequencing

For analysis of mutation on endogenous on‐target and off‐target sites, genomic DNA was extracted from cultured cells using the AccuPrep Genomic DNA Extraction Kit (Bioneer, Korea) according to the manufacturer's instructions. Potential off‐target sites were identified using Cas‐OFFinder (http://www.rgenome.net/cas‐offinder/), and those with 1–3 mismatches and DNA bulges were chosen for off‐target analysis. The extracted genomic DNA was amplified using a 2X Pfu‐PCR Master Mix (BN302‐50 h, BioFACT, Korea) for sequencing library generation. Libraries were sequenced using MiniSeq with a TruSeq HT dual index system (Illumina). The primers used are listed in Table S1, S2 (Supporting Information).

##### Animal Studies

All experimental procedures were approved and reviewed by the Korea Institute of Science and Technology (Approval No. KIST‐IACUC‐2022‐108 and IACUC‐2024‐012‐1). C57BL/6 and Balb/c mice (male, 8–10 weeks old, 18–30 g) (Charles River Laboratories, MA, USA) were utilized in this study. Mice were anesthetized with 2% isoflurane (Isothesia, Abbot Laboratories, IL, USA) with 1 L min^−1^ oxygen. To prepare an orthotopic mouse model of hepatocellular carcinoma, C57BL/6 mice were implanted with 5 × 10^6^ Hepa1c1c7 cells or Hepa1‐6 cells mixed 1:1 with Matrigel through an injection into the left lateral lobe of the liver using an insulin syringe (31 G, BD, NJ, USA) after exposing the liver via a subcostal incision of 5 mm or less. Following the procedure, the incision was closed with sutures, and the mice were monitored post‐operatively for recovery and tumor development.

##### In Vivo Imaging

CT studies were investigated using a micro‐CT imaging system (NanoScan PET/CT, Mediso, MA, USA) at the Center for Translational Imaging at Northwestern University. For phantom imaging, free tantalum pentachloride (TaCl_5_) and Ta‐doped UPSND were prepared to agar phantom models and investigated under 70 kVp of X‐ray. Hounsfield units of each sample were measured with RadiAnt DICOM viewer (version 2022.1, Medixant, Poland). For in vivo fluorescence monitoring, UPSND‐labeled with Cy7 was prepared. The orthotopic Hepa1c1c7 mice, after 4 weeks post‐inoculation, in each group (PBS and Cas9‐RNP@UPSND) were intravenously administered Cas9‐RNP@UPSND (concentration of UPSND, 8 mg mL^−1^) into the tail vein (*n* = 3). Bright‐field and fluorescence images of the mice were obtained periodically using in vivo fluorescence microscopy (IVIS Spectrum In Vivo Imaging System, PerkinElmer, CT, USA) using wavelength for Cy7 (745/800 nm). All acquired images were operated for pseudo‐coloring with fluorescence intensity.

##### In Vivo GPC3 Genome Editing in Hepa1‐6 Orthotopic HCC Models

Hepa1‐6 cells (2 × 10^6^ cells per mouse, in 50 μL of saline) were injected into the left lateral lobe of the liver.^[^ ^38^ ^]^ Briefly, mice were anesthetized, an incision was made, and the liver left lateral lobe was exposed. Tumor cells were injected to prevent the backflow. Incision was closed in two layers using 4–0 absorbable Vetacryl sutures (Ethicon, NJ, USA) and mice were given buprenorphine‐ER (extended release) (0.3 mg kg^−1^) (ZooPharm, WY, USA) for post‐operative care. After 7–10 days, the tumors were observed using Bruker 7.0 T ClinScan MRI T_2_‐weighted images (Bruker Biospin, Ettlingen, Germany). UPSND‐loaded Cas9‐RNP (Concentration of UPSND; 8 mg mL^−1^ in 100 μL in PBS) was directly intravenously injected 3 times every 3 days. Tumors were obtained on day 14. DNA was analyzed for T7 Endonuclease I (T7E1) assay and digested single cells were analyzed for the immune cell characterization using BD FACSymphony A5‐Laser Analyzer (BD Biosciences, NJ, USA).

##### T7E1 Assay

The target region of the gene was amplified by PCR using the primers mGPC3‐F (CAGGTAGCTGCGAGGAAACT) and mGPC3‐R (GCTCAACTTTGCCTGCACTT), along with Pfu DNA polymerase (BN302‐50 h, BioFACT, Korea). PCR cycling conditions were as follows: initial denaturation at 98 °C for 30 s, followed by 35 cycles of 98 °C for 10 s (denaturation), 57 °C for 15 s (annealing), and 72 °C for 20 s (extension), with a final extension at 72 °C for 2 min. The samples were then denatured at 95 °C for 5 min and gradually reannealed by cooling to 85 °C at 2 °C sec^−1^ and further cooling to 25 °C at 0.1 °C sec^−1^. The resulting PCR products were subjected to a T7E1 assay (M0302S, New England Biolabs, MA, USA). Briefly, 10 μL of PCR products were denatured at 95 °C for 5 min and reannealed to form heteroduplex DNA by slow cooling to 25 °C. The reannealed PCR products were then digested with 1 μL of T7E1 in a reaction mixture containing 2 μL of NEBuffer (New England Biolabs, MA, USA), and nuclease‐free water was added to bring the final reaction volume to 20 μL. The digestion was performed at 37 °C for 30 min, and the cleavage products were visualized by electrophoresis on a 10% acrylamide gel.

##### In Vivo GPC3 Genome Editing in HepG2‐bearing HCC Models

Mice were randomly divided into three groups as follows: control, Codrituzumab, and UPSND‐mediated GPC3 gene‐editing groups (*n* = 4). HepG2 cells (2 × 10^6^ cells per mice, in 100 μL of saline) or GPC3‐edited HepG2 cells (2 × 10^6^ cells per mice, in 100 μL of saline) were subcutaneously injected into the right flank of Balb/c mice. After 7 days, body weight and tumor volume (V) were monitored daily for 14 days and calculated with the following formula: V (mm^3^) = 0.5 × (width)^2^ × (length) (width ≤ length).

##### Immunohistochemistry

Tumors were harvested from the mice and fixed for 24 h in 4% paraformaldehyde. The tissue sections (5 μm) were stained with H&E, Ki67, and GPC3 (sc‐390 587, Santa Cruz Biotechnology, Inc., TX, USA). All slides were scanned using Nanozoomer 2.0‐HT slide scanner and observed using NDP.view2 software (Hamamatsu, Shizuoka, Japan).

##### Immune Cell Characterization Using Flow Cytometry

Tumor tissue cells and tumor‐draining lymph node immune cells were dissected and homogenized by the rubber part of a syringe and were sieved to exclude the extra tissue samples using a cell strainer (40 μm, Corning, NY, USA). Erythrocytes were lysed using red blood cell lysis buffer (Biolegend, CA, USA) for 3‐5 min at RT. The number of purified cells was counted by Countess I (Thermofisher, MA, USA), and 1 × 10^7^ cells were stained with indicated antibodies to evaluate the immune cell activation. The antibodies used were listed in Table S3, Supporting Information. All flow cytometry analysis was performed using BD FACSymphony A5‐Laser Analyzer (BD Biosciences, NJ, USA). Data analysis was performed using FlowJo software (TreeStar) (BD Bioscience, NJ, USA).

##### Statistical Analysis and Reproducibility

All images were obtained from at least three independent experiments with similar results. Also, all data were shown mean corrected values ± s.d. of at least three independent experiments unless otherwise stated. Data were analyzed using Prism software version 10.0 (GraphPad, CA, USA). Graphical data are presented as means ± s.d. Statistical significance of differences between two groups was determined using student's unpaired t‐test or one‐way analysis of variance. P‐values were considered statistically significant following stars: *p < 0.05, **p < 0.01, ***p < 0.001, ****p < 0.0001; ns, not significant.

## Conflict of Interest

The authors declare no conflict of interest.

## Author Contributions

**Sanghee Lee**: Conceptualization (equal); Data curation (lead); Formal analysis (lead); Investigation (lead); Methodology (lead); Writing—original draft (equal); Writing—review and editing (equal). **Sian Lee**: Formal analysis (supporting); Investigation (equal); Methodology (equal); Validation (equal); Visualization (equal); Writing—review and editing (supporting). **Hyojin Lee**: Funding acquisition (supporting); Project administration (supporting); Resources (supporting); Supervision (supporting); Writing—review and editing (supporting). **Seongchan Kim**: Conceptualization (equal); Data curation (equal); Formal analysis (equal); Funding acquisition (supporting); Investigation (equal); Methodology (supporting); Resources (supporting); Supervision (supporting); Validation (equal); Visualization (equal); Writing—original draft (supporting); Writing—review and editing (equal). **Dong‐Hyun Kim**: Conceptualization (lead); Data curation (supporting); Formal analysis (supporting); Funding acquisition (lead); Investigation (equal); Methodology (supporting); Project administration (lead); Resources (lead); Supervision (lead); Validation (supporting); Visualization (supporting); Writing—original draft (lead); Writing—review and editing (lead).

## Supporting information

Supplementary Material

## Data Availability

The data that support the findings of this study are available from the corresponding author upon reasonable request.
