# Supplementary material for: Therapeutic Glypican‐3 CRISPR Genome‐Editing Using UltraLarge Porous Silica Nano‐Depot for the Treatment of Hepatocellular Carcinoma
Source: Small Sci. 2024 Nov 21;5(4):2400447. doi: 10.1002/smsc.202400447 (PMC12245085; doi:10.1002/smsc.202400447)
Supplement: Supplementary file 1 — Supplementary Material [file SMSC-5-2400447-s001.pdf]

Supporting Information

**Therapeutic Glypican-3 CRISPR Genome-editing Using Ultra-large Porous Silica Nano-depot for The Treatment of Hepatocellular Carcinoma**

*Sanghee Lee, Sian Lee, Hyojin Lee*, Seongchan Kim*, and Dong-Hyun Kim**

Dr. S. Lee, Prof. S. Kim, Prof. D.-H. Kim
Department of Radiology, Feinberg School of Medicine
Northwestern University, Chicago, IL 60611, USA
E-mail: dhkim@northwestern.edu

S. Lee, Dr. H. Lee
Biomaterial Research Center, Biomedical Research Institute
Korea Institute of Science and Technology (KIST)
Seoul 02792, Republic of Korea
E-mail: hyojinlee@kist.re.kr

Prof. S. Kim
College of Pharmacy and Research Institute of Pharmaceutical Sciences
Gyeongsang National University
Jinju, Gyeongsangnam-do 52828, Republic of Korea
E-mail: seongchan.kim@gnu.ac.kr

Prof. D.-H. Kim
Robert H. Lurie Comprehensive Cancer Center
Northwestern University, Chicago, IL 60611, USA

Department of Biomedical Engineering
McCormick School of Engineering
Northwestern University, Evanston, IL 60208, USA

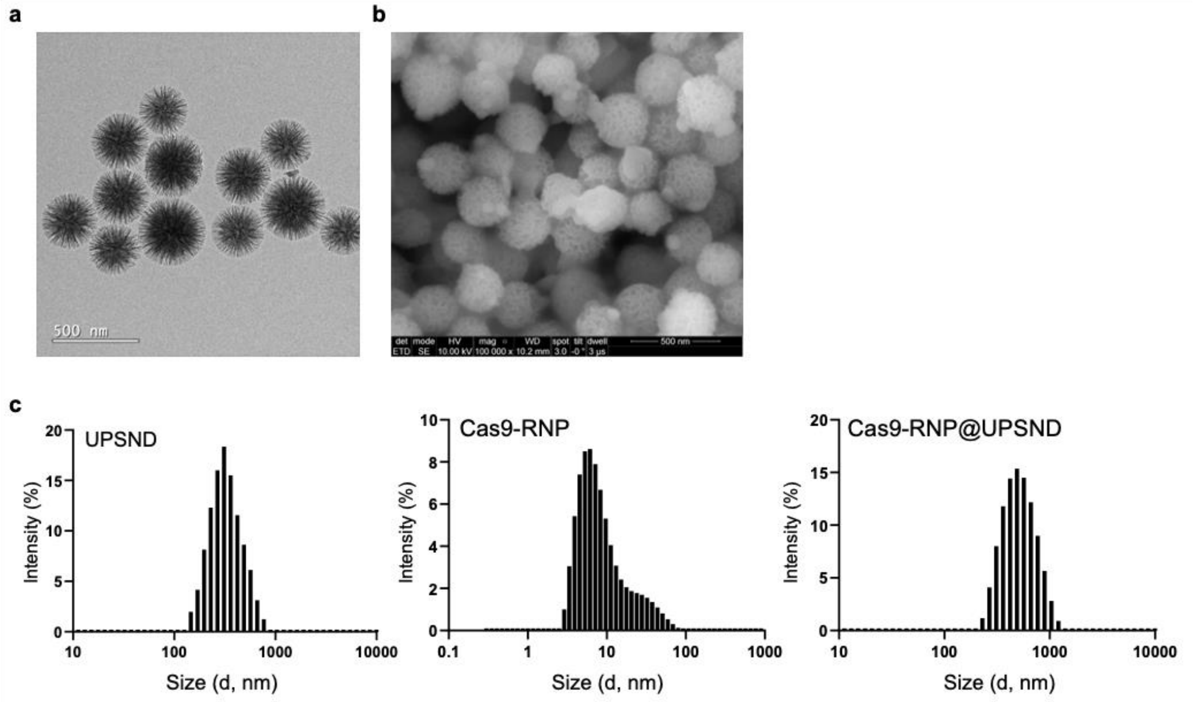


**Figure S1.** Preparation of an ultra-large porous silica nano-depot (UPSND) and Cas9-ribonucleoprotein (RNP). a) Transmission electron microscopy (TEM) and b) scanning electron microscopy (SEM) image of UPSND. Scale bar is 500 nm. c) Dynamic light scattering (DLS) data of UPSND, Cas9-RNP, and Cas9-RNP@UPSND to determine the size distribution profile.

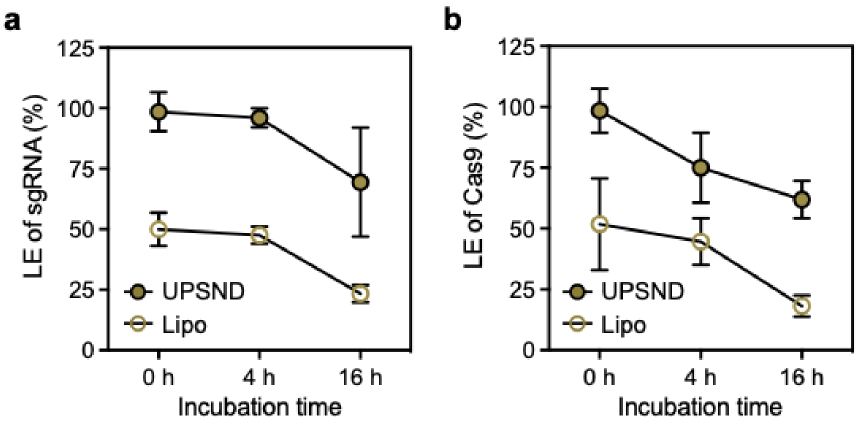


**Figure S2.** Characterization of UPSND and Cas9-RNP. Loading efficiency (LE) and released profiles of a) sgRNA and b) Cas9 from UPSND-loaded and Lipo-loaded Cas9-RNP in PBS at 37$^{o}$C over time ($n$ = 4).

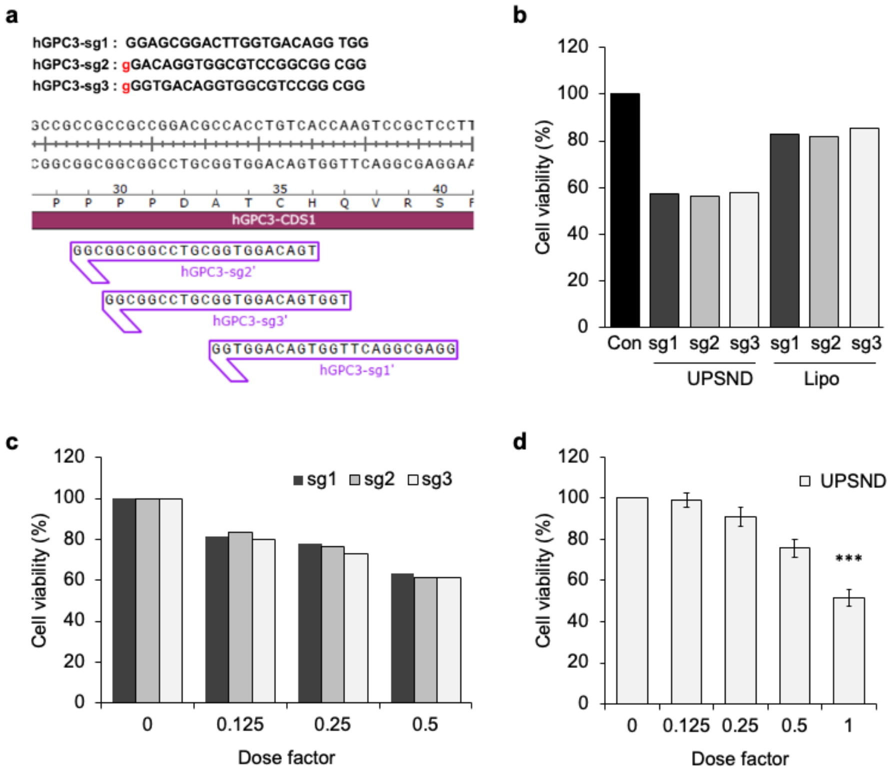


**Figure S3.** Design and selection of sgRNA candidates targeting GPC3 in mouse HCC. a) In-silico analysis identified potential sgRNA sequences and evaluated off-target effects. Three sgRNA candidates were selected based on optimal targeting efficiency and minimal off-target potential. b) Cell viability following Lipo- or UPSND-mediated GPC3 gene editing with three sgRNA candidate ($n$ = 4). c) Cell viability following UPSND-mediated GPC3 gene editing at the various concentration of hGPC3-sg3 with a constant UPSND-to-sgRNA ratio ($n$ = 4). d) Cell viability following UPSND-mediated GPC3 gene editing at the various UPSND-to-sgRNA ratios ($n$ = 8).

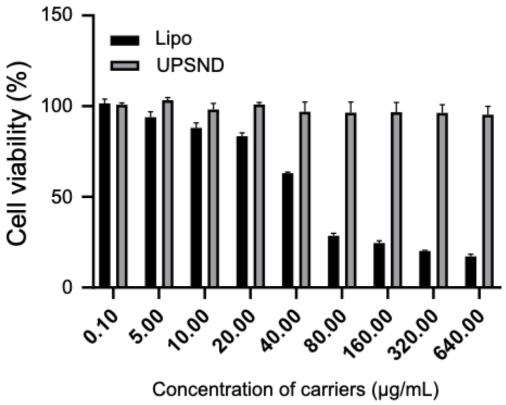

**Figure S4.** Cell viability of UPSND and Lipo in HepG2 cells.

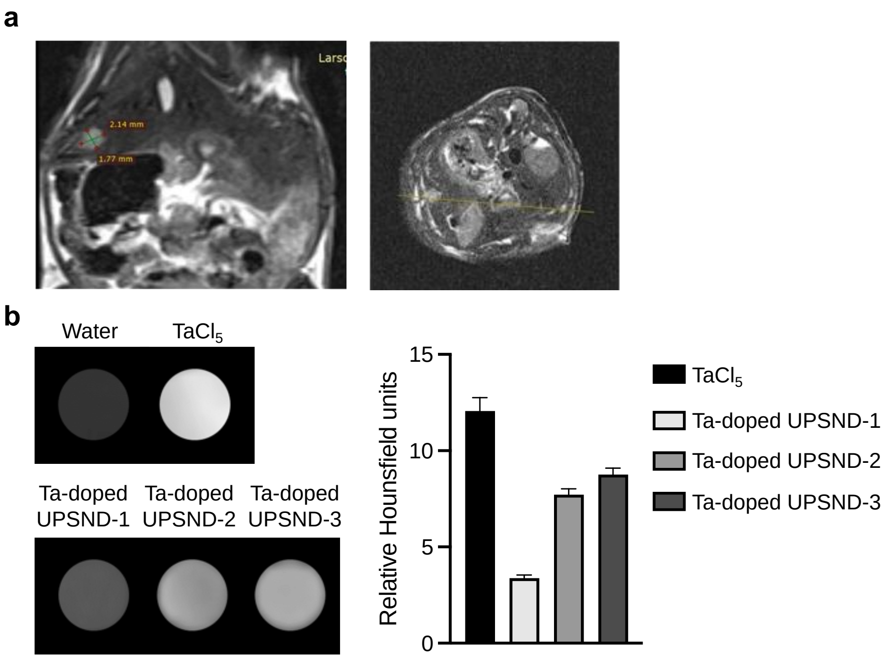


**Figure S5.** Hepa1-6 orthotopic murine HCC models and the potential for computed tomography (CT) image-guided theragnostics. a) Tumors were observed using $T_2$-weighted magnetic resonance imaging. b) CT phantom images of tanalum (Ta)-doped UPSND at various concentration (1, 2 mg $mL^{-1}$; 2, 5 mg $mL^{-1}$; 3, 10 mg $mL^{-1}$; water served as a negative control and free tantalum pentachloride ($TaCl_5$) served as a positive control). Relative Hounsfield units were calculated based on the value of water ($n$ = 3).

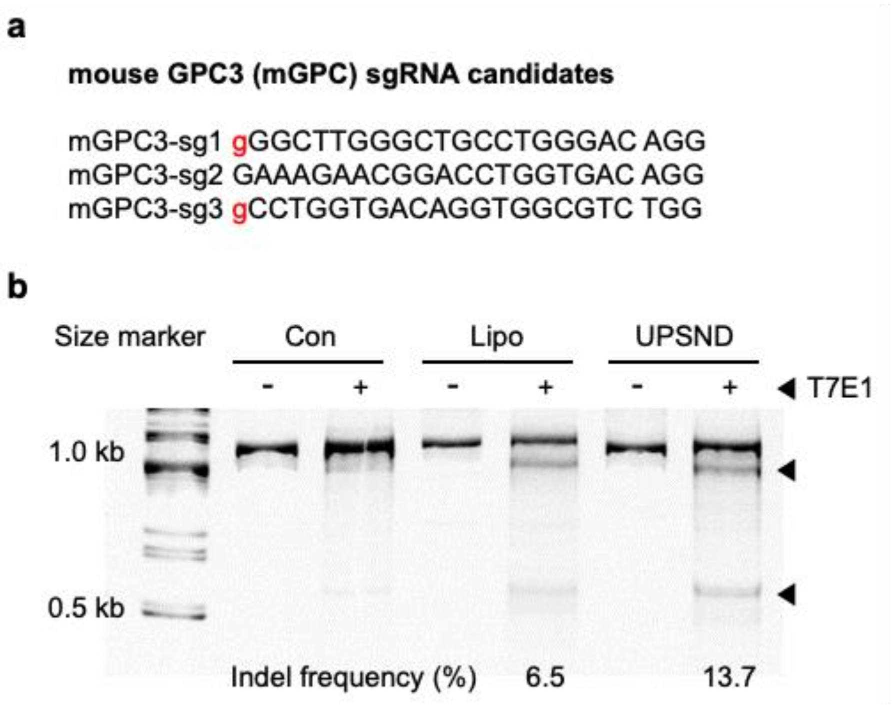


**Figure S6.** GPC3 gene deletion efficiency in animal experiments. a) In-silico analysis identified potential mouse GPC3 (mGPC3)-sgRNA sequences. Three sgRNA candidates were selected based on optimal targeting efficiency and minimal off-target potential. b) T7E1 indel analysis of GPC3 gene editing in Hepa1-6 HCC tumors. Tumors were harvested on day 3 post-gene editing.

**Table S1.** List of primers used to amplification of genomic DNA for targeted deep sequencing (seq) (F, forward primer; R, reverse primer).

| Target gene | | DNA oligomer (5'→3') |
|---|---|---|
| hGPC3 | sgRNA | gGGTGACAGGTGGCGTCCGG |
| | Deep seq primer 1st F | TGCGAGGAAACTTTTGCAGC |
| | Deep seq primer 1st R | CTCCCTCCCTCAGTAGACCC |
| | Deep seq primer 2nd F | ACACTCTTTCCCTACACGACGCTCTTCCGATCTCTCTCCTAGCTCCCTGCGAA |
| | Deep seq primer 2nd R | GTGACTGGAGTTCAGACGTGTGCTCTTCCGATCTCTGGCACGGGAGTTTCTGG |
| mGPC3 | sgRNA | gCCTGGTGACAGGTGGCGTCTGG |
| | Deep seq primer 1st F | CAGGTAGCTGCGAGGAAACT |
| | Deep seq primer 1st R | CTCTGGCTTGCACAGTTCCT |
| | Deep seq primer 2nd F | ACACTCTTTCCCTACACGACGCTCTTCCGATCTTCCGGCTCTTATTGCCACTC |
| | Deep seq primer 2nd R | GTGACTGGAGTTCAGACGTGTGCTCTTCCGATCTATGCTCAAGGGACCCCTACT |

**Table S2.** List of primers used for amplification of genomic DNA for targeted deep sequencing.

| Human GPC3 | ID (OT) | Sequence | Mismatch | Locus |
|---|---|---|---|---|
| On-target | | gGGTGACAGGTGGCGTCCGG | 0 | chX : 133985339 |
| Off-target (OT) | OT-1 | GGGaGACAGGTGGgGTgCGGGGG | 3 | chr8 : 1825276 |
| | OT-2 | GGGTGACtGGTGGCGTtaGGGGG | 3 | chr8 : 51681245 |
| | OT-3 | GGGTGACAGGTGGCcTCtGtGGG | 3 | chr7 : 32259787 |
| | OT-4 | GGGaGACAGGTGGCcTCCaGAGG | 3 | ch12 : 109552589 |
| | OT-5 | GGGTGACcGGTGGCcTCCtGGGG | 3 | chr17 : 21141196 |
| | OT-6 | GGGTGgCAGGaGGCGTCCaGCGG | 3 | chr16 : 2115489 |
| | OT-7 | GGGTGgCAGGaGGCGTCCaGCGG | 3 | chr16 : 14921392 |
| | OT-8 | GGGTGACAGcaGGtGTCCGGAGG | 3 | chr16 : 81589989 |
| | OT-9 | GGGTGgCAGGaGGCGTCCtGGGG | 3 | chr19 : 3047348 |
| | OT-10 | GGGTGACAGGgGGCcTCaGGTGG | 3 | chr19 : 7615846 |

| Mouse GPC3 | ID (OT) | Sequence | Mismatch | Bulge Type – Size | Locus |
|---|---|---|---|---|---|
| On-target | | gCCTGGTGACAGGTGGCGTCTGG | 0 | DNA – 1 | chrX : 52613694 |
| Off-target (OT) | OT-1 | GaCTGGTGACAGGTGGGgGaCTGGTGG | 3 | DNA – 1 | chr15 : 73175085 |
| | OT-2 | GCCTGGTGACAGGTGaCAGaCTaGGGG | 3 | DNA – 1 | chr5 : 36234418 |
| | OT-3 | GCCgGGTGcCAGGTGGCCGTgTGGTGG | 3 | DNA – 1 | chr9 : 110428976 |
| | OT-4 | GACCTGGTGACAGGTGGCGTCTGGAGG | 3 | DNA – 1 | chrX : 52613693 |
| | OT-5 | GCCATGGTGACAGGaGGtGTCTaGAGG | 3 | DNA – 1 | chr11 : 114004884 |
| | OT-6 | GCCTGGTGAtCAGGTGGCGgCaGGGGG | 3 | DNA – 1 | chr10 : 81275892 |
| | OT-7 | GCCTGGTGACAGGTGcCaTGCTGcAGG | 3 | DNA – 1 | chr3 : 152722964 |

**Table S3.** List of antibodies, proteins, and enzyme-linked immunosorbent assay (ELISA) kits used in this study.

| No | Product name | Cat No. | Clone | Dilution ratio | Manufacture |
|---|---|---|---|---|---|
| 1 | Human Glypican 3 ELISA Kit | DGLY30 | - | - | R&D systems |
| 2 | Mouse Glypican 3 ELISA Kit | EKN53405 | - | - | BIOMATIK |
| 3 | Wnt5a/b (C27E8) Rabbit mAb | 2530 | - | 1:1000 | Cell Signaling Technology |
| 4 | LRP6 (C47E12) Rabbit mAb | 3395 | - | 1:1000 | Cell Signaling Technology |
| 5 | Phospho-LRP6 (Ser1490) Antibody | 2568 | - | 1:1000 | Cell Signaling Technology |
| 6 | Anti-beta Catenin non-phospho S37/T41 antibody [EPR23969-131] | ab246504 | - | 1:1000 | abcam |
| 7 | LATS1 (C66B5) Rabbit mAb | 3477 | - | 1:1000 | Cell Signaling Technology |
| 8 | Phospho-LATS1 (Ser909) Antibody | 9157 | - | 1:1000 | Cell Signaling Technology |
| 9 | YAP/TAZ (D24E4) Rabbit mAb | 8418 | - | 1:1000 | Cell Signaling Technology |
| 10 | Phospho-YAP (Ser127) (D9W2I) Rabbit mAb | 13008 | - | 1:1000 | Cell Signaling Technology |
| 11 | Phospho-YAP (Ser397) (D1E7Y) Rabbit mAb | 13619 | - | 1:1000 | Cell Signaling Technology |
| 12 | Anti-rabbit IgG, HRP-linked Antibody | 7075 | - | 1:1000 | Cell Signaling Technology |
| 13 | beta Actin Polyclonal Antibody | PA5-85291 | - | 1:5000 | Invitrogen™ |
| 14 | BV605 Rat Anti-Mouse CD45 | 563053 | 30-F11 | 1:100 | BD Bioscience |
| 15 | BV510 Anti-mouse CD3 Antibody | 100234 | 17A2 | 1:100 | Bio legend |
| 16 | APC Rat Anti-Mouse CD4 | 553051 | RM4-5 | 1:100 | BD Bioscience |
| 17 | FITC Rat Anti-Mouse CD8a | 553031 | 53-6.7 | 1:100 | BD Bioscience |
| 18 | APC-Cy™7 Rat Anti-Mouse CD25 | 557658 | PC61 | 1:100 | BD Bioscience |
| 19 | PE-CF594 Rat Anti-Mouse Foxp3 | 562466 | MF23 | 1:100 | BD Bioscience |
| 20 | PE-CF594 Rat Anti- Ki-67 | 567120 | B56 | 1:100 | BD Bioscience |
| 21 | Codrituzumab | HY-P99013 | - | - | MedChemExpress |
| 22 | PE-Labeled Human Glypican 3 Protein | GP3-HP2E3 | - | - | Acro Biosystems |
| 23 | glypican-3 Antibody (F-3) | sc-390587 | - | 1:50 | Santa cruz |
| 24 | BV421 Rat Anti-Mouse CD274 | 564716 | MIH5 | 1:100 | BD Bioscience |
